# Effectiveness of a Gamified Mobile App in Enhancing Treatment Adherence for Children With Amblyopia: Explorative Study

**DOI:** 10.2196/60309

**Published:** 2025-10-28

**Authors:** Bo Liu, Yisheng Fan, Meng Xu, Fangyuan Chang, Yue Shi, Zhao Liu

**Affiliations:** 1 College of Design, Shanghai Jiaotong University, 800 Dongchuan Road, Minhang District, Shanghai, 200240, China, 86 021-54742134

**Keywords:** amblyopia, child health care innovation, emotional cognition, gamified therapy, mobile health apps, pediatric eye care, randomized controlled trial, serious game, treatment adherence, visual rehabilitation

## Abstract

**Background:**

Amblyopia is the leading cause of visual impairment in children worldwide. The predominant clinical treatment, occlusion therapy, is marred by poor adherence, often attributed to the physical discomfort and social stigma associated with eye patching. Adjunct digital visual trainings have not consistently sustained patient engagement due to their repetitive nature, thereby compromising their efficacy.

**Objective:**

This study aimed to evaluate the effectiveness of a gamified mobile app designed to increase treatment adherence among children with amblyopia by making the therapeutic process more engaging and accessible within home settings.

**Methods:**

An exploratory study was conducted, commencing with qualitative interviews and questionnaires to explore the barriers to traditional treatment adherence. This formative research informed the development of a gamified mobile app, which was shaped by cognitive appraisal theory to address identified emotional and psychological needs, potentially impacting adherence. The subsequent quantitative phase utilized a randomized controlled trial involving 34 children with amblyopia who were aged 7‐10 years and recruited from a local primary school. These participants were randomly assigned to either the intervention group, which used a novel gamified mobile app developed by our team, or the control group, which utilized another commercially available mobile app. Both groups engaged with their respective apps in a home environment. The 8-item Morisky Medication Adherence Scale was adapted to measure treatment adherence.

**Results:**

Over the 4-week trial, 34 children aged 7‐10 years with amblyopia were enrolled and randomized into 2 groups: intervention (n=18) and control (n=16). Children in both the intervention and control groups engaged daily for 20 minutes at home, using mobile apps designed for visual rehabilitation. The intervention group (n=18) achieved a significantly higher mean adherence rate (mean 6.56, SD 1.06) on the Morisky Medication Adherence Scale compared to the control group (n=16; mean 5.01, SD 1.22; *P*<.001). Thematic analysis of the design process revealed that integrating cognitive appraisal theory effectively enhanced emotional engagement and adherence.

**Conclusions:**

The integration of cognitive appraisal theory into the design of a gamified mobile app for amblyopia treatment has shown to significantly improve adherence among children.

## Introduction

### Background

Amblyopia, the most prevalent cause of visual impairment in children, affects a significant number of young individuals worldwide [1-4]. It leads to deficits in visual acuity, spatial perception, and visual processing, making early intervention essential [5-11]. Currently, the primary treatment involves occlusion therapy—patching the healthy eye to stimulate the amblyopic one [1,5,12,13]. However, adherence to this method is low, often under 50%, due to psychological stress, discomfort, and inadequate parental support, resulting in suboptimal treatment outcomes [14-20]. To overcome these challenges, digital therapy has emerged as a promising supplement to traditional methods [1,21,22]. Visual training tasks delivered through digital platforms, such as contrast detection or letter recognition games, can stimulate the amblyopic eye while engaging children in interactive, structured activities [23-27]. Studies suggest that incorporating gamification into such tasks may enhance participation and adherence [7,27-33]. Despite this promise, many digital therapies remain ineffective due to repetitive content, limited personalization, and poor user engagement [1,9,21,22,27,28,32-34]. Reliance on bulky equipment or clinical settings often adds logistical burdens for families, whereas mobile-based solutions offer portability, accessibility, and alignment with children’s behavioral tendencies, making them more suitable for home-based rehabilitation [8,21,35,36].

Within this landscape, evidence indicates that family-centered care models, particularly those integrating interactive game designs, can significantly enhance adherence and therapeutic outcomes among children with amblyopia [7,21,22]. Gamified approaches that incorporate emotional support and social feedback effectively address resistance to eye patching and repetitive training, thereby improving adherence [37]. Features such as immediate feedback and reward systems within gamified interventions help sustain interest and reinforce habitual therapeutic behaviors over time [38,39]. Integrating cartoon-based education and visual feedback into game design has been shown to markedly boost short-term adherence in children undergoing amblyopia care [37,40,41]. Beyond short-term engagement, gamification can foster long-term rehabilitative habits through immersive and layered situational experiences that scaffold ongoing participation [42]. An increasing body of research is now investigating digital interventions to improve adherence and therapeutic outcomes in pediatric amblyopia, and the current market already offers mobile health apps that combine visual stimulation with simple games to capture children’s attention [43].

### Rationale

Yet, a critical gap persists: the emotional and psychological needs of children with amblyopia are often overlooked, and while advanced interventions like virtual reality and binocular therapies claim to improve engagement, few solutions are designed with explicit attention to children’s cognitive and emotional experiences, which may compromise long-term adherence and therapeutic effectiveness [24,36]. Many existing mobile apps also lack diversity in interactivity and feedback mechanisms, limiting their ability to sustain engagement over time despite providing visual stimulation that can improve adherence [1,34,44]. Research shows that when content richness or dynamic feedback is insufficient, engagement levels diminish, underscoring the need for gamified therapeutic solutions that integrate greater interactivity and real-time feedback to better support home-based rehabilitation [1,34,44]. Moreover, evidence on the long-term vision improvement benefits of digital interventions remains limited, and concerns persist about potential negative effects such as visual fatigue and myopia from prolonged use of small-screen devices, highlighting the need for further research to define their role and impact in amblyopia rehabilitation [45].

To address these limitations, we conducted an exploratory study using qualitative interviews and questionnaires to identify key adherence barriers, and we developed a mobile-based gamified intervention informed by cognitive appraisal theory to target the emotional and behavioral needs of users with pediatric conditions. The intervention was integrated into standard visual training routines and evaluated through a randomized controlled trial (RCT) to test whether a theory-informed, emotionally intelligent design could improve adherence and engagement in home settings.

### Theoretical Framework: Cognitive Appraisal Theory

In both psychology and marketing, emotional states are recognized as critical drivers of effective individual actions. Among the various theories exploring the relationship between emotion and cognition, Lazarus’s cognitive appraisal theory has garnered significant attention. This theory posits that emotions arise from an individual’s subjective evaluation of an event, meaning emotions are the result of appraisals, which subsequently influence behavior [46].

In specific applications, cognitive appraisal theory has been utilized to explain the emotional states elicited by users’ appraisals of products, services, environments, or events [47,48]. It has been widely adopted in fields such as tourism and consumer behavior to explore the comprehensive interplay of cognition, emotion, and behavior triggered by interactions with particular events or physical environments [49]. Notably, research related to entertainment games has demonstrated that players’ cognitive appraisals of video games can elicit both positive and negative emotional states [50]. Kim et al further argued that compared to other products or services, video games are often perceived as leisure activities from an emotional appraisal perspective [51]. This suggests that users prioritize enjoyment and pleasure [50].

In the context of our study, cognitive appraisal theory offers a valuable framework for understanding and integrating users’ emotional responses into the design of gamified interventions. By leveraging this theory, we can systematically guide the emotional responses of children toward specific products or services, aligning with their psychological and emotional needs. Integrating cognitive appraisal theory into our intervention design allows us to address adherence challenges by enhancing emotional engagement and fostering positive emotional states, which are essential for the success of gamified treatments for amblyopia.

Table 1 summarizes the five core dimensions—relevance, valence, likelihood, agency, and coping potential—and their definitional criteria as established in the appraisal literature [46,48,52-54]. Definitions emphasize how appraisals of goal significance, expected outcomes, perceived control, and coping capacity shape emotions that, in turn, drive behavior [46,48,52-54]. Prior work shows that emotional support improves adherence to amblyopia therapy in children, with enhanced relevance and positive valence fostering engagement and role-play strengthening agency and self-efficacy [37]. A multidimensional, appraisal-guided approach has been reported to increase user engagement and positive participation, with particularly strong effects among child users in serious game contexts [39].

**Table 1.** Criteria for dividing dimensions of cognitive evaluation theory.

Dimensions	Criteria
Relevance	The anticipation of achieving a more valuable goal or outcome can result in a heightened emotional response. This is closely linked to the experience of interest [52,53], which is a typical reaction to situations that are subjectively significant and elicit high levels of attentional activity. This, in turn, increases the likelihood of responding effectively to events and evokes strong positive emotions, such as enjoyment [54].
Valence	Individuals are more likely to experience positive emotions, such as happiness or gratitude, when they perceive an experience as related to a positive goal and moving them closer to that desired goal [54].
Likelihood	The likelihood dimension assesses the probability of a situation leading to a specific outcome, identifying associated emotions such as hope, fear, or anxiety [46,54].
Agency	Consider the influence of the user, others, or the environment/objects on the outcome of the situation, as well as factors related to controllability, in the evaluation of the agent [48].
Coping potential	Coping potential is the perceived ability to manage or change a situation and is categorized into 3 main groups: benign-positive, neutral, and stressful [46].

### Study Aim

Guided by cognitive appraisal theory and the growing evidence for gamified digital interventions, this study developed and evaluated a mobile serious game to address emotional and psychological drivers of adherence in pediatric amblyopia care. The aim was to support sustained, home-based rehabilitation by personalizing mechanics, narratives, and feedback to children’s appraisal processes, thereby improving treatment adherence in everyday settings. We tested this approach in an RCT with children aged 7‐10 years completing daily home training, aligning with best practices for interventional evaluation in digital health. The primary outcome was treatment adherence at 4 weeks measured with an adapted 8-item Morisky Medication Adherence Scale (MMAS-8), and secondary outcomes included user experience dimensions (eg, attractiveness, novelty, usability) and qualitative engagement mapped to appraisal dimensions. We hypothesized that an appraisal-informed, child-centric design would help overcome conventional barriers to occlusion and repetitive training and enhance engagement in pediatric vision care.

## Methods

### Study Design

We used a 3-stage mixed methods design comprising a qualitative needs assessment (Stage 1), an expert-informed design (Stage 2), and a parallel-group RCT (Stage 3) to develop and evaluate a gamified mobile intervention for pediatric amblyopia rehabilitation. The qualitative components were conducted and reported in line with COREQ (Consolidated Criteria for Reporting Qualitative Studies) and employed inductive thematic analysis with dual coding in NVivo 14 (developed by QSR International) to enhance rigor and credibility. The interventional RCT was designed and reported according to CONSORT-EHEALTH (Consolidated Standards of Reporting Trials of Electronic and Mobile Health Applications and Online Telehealth) to ensure transparency and reproducibility in digital health trials. Insights from Stages 1‐2 identified key adherence barriers and specified design levers grounded in cognitive appraisal theory to inform the gamified app’s mechanics, narrative, and feedback. The primary outcome for Stage 3 was treatment adherence at 4 weeks measured with an adapted MMAS-8, and secondary outcomes included User Experience Questionnaire (UEQ) scores and posttrial interviews mapping children’s emotional engagement to appraisal dimensions. The trial was registered at ClinicalTrials.gov (NCT06372548) and created to cover the entire study, including foundational qualitative and design phases.

### Ethical Considerations

This study was approved by the Institutional Review Board of the Eye & ENT Hospital of Fudan University (H20230365I). Written parental consent was obtained for all minors, and child assent was acquired using a plain-language cartoon booklet and an assent form completed (signature or fingerprint) prior to any study procedures. Participation was discontinued immediately if a child indicated distress or refusal, in accordance with established child-safeguarding protocols. All data were deidentified and stored on encrypted hospital servers with dual backup, with a retention policy of 5 years before secure destruction. Participants received a stationery set and ¥20 (approximately US $2.80) upon completion as compensation for time and effort.

The ClinicalTrials.gov registration (NCT06372548) was submitted following completion of the initial qualitative phase but prior to the recruitment and enrollment of participants into the randomized controlled intervention. Importantly, the registration occurred before any RCT participant was recruited or randomized, ensuring that all interventional procedures and outcomes were prospectively registered.

### Stage 1: Qualitative Needs Assessment

#### Participants and Recruitment

We used purposive, maximum-variation sampling to capture a diverse range of experiences and perspectives. The inclusion criteria included (1) parent or legal guardian of a child aged 6‐10 years diagnosed with amblyopia, (2) pediatric ophthalmologist with ≥3 years of clinical experience, or (3) child meeting diagnostic criteria who could assent with parental help. The exclusion criteria included inability to communicate in Mandarin or refusal to be recorded. Parents were approached during clinic visits; clinicians were invited by email; children were recruited via clinician referral. In total, 8 ophthalmologists, 22 parents (8 families), and 6 children participated. The sample size was guided by information power, and theoretical saturation was reached by the sixth parent interview, as no new codes or themes emerged, indicating sufficient coverage of core concepts.

#### Interview Guide

Semistructured interview guides were pilot-tested with 2 parents and 1 clinician, then refined for clarity and relevance. The interview topics covered barriers to occlusion therapy, child motivation, game preferences, and parental support. The interview outlines for parents and children are available in MultimediaAppendices 1  2.

#### Data Collection

All interviews were conducted by 2 trained researchers (a female design graduate student and a male MSc research assistant in ophthalmology) in private clinic rooms (for parents or clinicians) or a quiet play area (for children). Each interview lasted 40‐60 minutes, was audio-recorded, transcribed verbatim, and securely backed up on encrypted drives. Member-checking was used for credibility: summary transcripts were sent to 4 participants to confirm the accuracy and resonance of identified themes.

#### Analysis and Credibility

Transcripts and questionnaires were analyzed using inductive thematic analysis in NVivo 14: familiarization with data, initial coding (open coding), theme development (axial and selective coding), theme review, theme definition/naming, and reporting [55]. At least 2 coders independently performed each coding round; disagreements were discussed with a third coder until consensus was reached, resulting in high intercoder reliability (κ=0.82). At least 2 anonymized, supportive quotations per theme are provided in the *Results* section for confirmability.

Reflexivity and trustworthiness were prioritized throughout analysis. Researchers maintained a reflexive diary to document positionality, preconceptions, and their influence on data interpretation. Confirmability was established through audit trails and member checking. Dependability was ensured via transparent documentation of all analytic steps and coder discussions. Credibility was strengthened by participant feedback; thick description; and triangulation between parent, child, and clinician interviews. Transferability is enhanced by detailed reporting of sample characteristics and recruitment context, enabling readers to assess relevance to other settings.

### Stage 2: Expert-Informed Design Process

#### Expert Panel and Sampling

An expert panel was assembled using purposive sampling across hospital, academic, and design networks to capture diverse knowledge relevant to pediatric amblyopia treatment and gamified intervention development. A total of 6 experts were recruited, including 3 pediatric ophthalmologists (each with at least 5 years of clinical experience) and 3 game designers (each with a minimum of 5 years of experience in serious games or health-related gamification). The inclusion criteria comprised verified domain expertise and willingness to engage in 2 rounds of structured consultation.

#### Round 1 Interviews and Design Mapping

In Round 1, each expert participated in a 45‐ to 60-minute Zoom interview focused on operationalizing the 5 dimensions of cognitive appraisal theory (relevance, valence, likelihood, agency, coping potential) within the concrete mechanics, narrative structures, feedback modalities, and visual styles suitable for pediatric rehabilitation. Interview questions, refined following pilot feedback, are available in Multimedia Appendix 3. All interviews were audio-recorded, transcribed verbatim, and thematically coded by 2 independent coders in NVivo 14 following an inductive-deductive hybrid approach; intercoder reliability for theme extraction was κ=0.79. The team used reflexive diaries to minimize bias, ensuring that both clinical and design perspectives were adequately represented. Coded insights from all experts were synthesized into a design-decision matrix that mapped cognitive appraisal dimensions to actionable game features. This matrix guided ideation of low-fidelity paper sketches, followed by a high-fidelity Figma digital prototype (Prototype 1) that reflected prioritized features and narrative components identified in Round 1.

#### Round 2 Consensus and Prototype Refinement 

In Round 2, experts were provided asynchronous access to Prototype 1 and asked to rate each proposed feature on a 7-point Likert scale for relevance and feasibility. Consensus was defined a priori as at least 80% agreement per domain and item. All items met or exceeded this threshold after minor revision based on expert feedback, resulting in a finalized intervention prototype (Prototype 2) used in the subsequent RCT. Reflexivity was maintained during consensus scoring, and disagreements were reconciled through written clarification and reevaluation to ensure the transferability of core design principles to clinical practice and child end-user contexts.

### Stage 3: Randomized Controlled Trial

#### Participants and Recruitment

Children aged 7-10 years with refractive, strabismic, or mixed amblyopia were recruited via school outreach and clinic referrals. The inclusion criteria were a confirmed diagnosis, ability to follow the training with parental support, and access to a mobile device at home. The exclusion criteria included severe amblyopia (best corrected visual acuity≤0.2), other ocular or neurological comorbidities, and prior exposure to digital amblyopia therapy. Based on pilot data (expected mean difference 1.5 on MMAS-8, SD 1.2; Cohen *d*=1.25), 15 participants per arm were required to achieve 90% power at *α*=.05 (2-tailed). We therefore enrolled 40 participants to mitigate risk of loss to follow-up and randomized after consent or assent.

#### Randomization, Concealment, and Masking

An independent statistician generated a computer-based permuted-block sequence (block size=4). Allocation was concealed using sequentially numbered, opaque, sealed envelopes. Outcome assessors and the statistician were blinded to group assignment.

#### Procedures

After written parental consent and child assent, participants completed a baseline enrollment visit during which we verified eligibility and collected demographic variables on a standardized case report form: age (in years), sex (male/female), and school grade (3rd-5th), as well as device availability for home use; demographic sources were parent report at enrollment using a structured checklist. Participants were then randomized to the intervention (our gamified app, “Find You! Cure My Animal Friends”) or the control (a widely used commercial visual-training app) and received a standardized orientation, including a brief instructional video for parents and a printed quick-start guide covering daily use, patching alongside play as clinically indicated, and troubleshooting. Families were instructed to complete home-based training for 20 minutes per day over 4 consecutive weeks; no additional clinical care was altered for either group during the study period. To support consistency, parents were asked to supervise session start/stop and encourage completion at roughly the same time each day.

#### Attrition and Analysis Set

During data collection, 6 participants (3 per arm) withdrew (reasons reported to the study team: lack of reliable home internet, scheduling conflicts, or unrelated illness). Data from these participants were not used in the analysis. The final analytic sample was N=34 (intervention n=18; control n=16).

#### Postintervention Assessment

The primary outcome was treatment adherence, assessed with an adapted MMAS-8 (licensed from Morisky LLC; see Multimedia Appendix 4 for item wording and scoring) [56,57]. Within the adaptation, medication-specific terms were reworded to “vision training” and “eye patch” to ensure contextual relevance for pediatric amblyopia. Comprehensibility and content validity were confirmed via cognitive interviews with 5 parent-child dyads prior to trial launch; pilot administration with n=20 yielded Cronbach *α*=.83, supporting internal consistency. The MMAS-8 was administered at the end of week 4 with parental assistance, and total scores were classified into low, medium, or high adherence according to developer guidance.

Secondary outcomes included the UEQ (26 items across 6 dimensions, see Multimedia Appendix 5) and postintervention semistructured interviews with children and parents (20‐40 min, conducted via Zoom, see Multimedia Appendix 6). These interviews explored emotional and behavioral engagement with the intervention, thematically mapped to cognitive appraisal theory dimensions (relevance, valence, likelihood, agency, and coping potential).

All interviews were audio-recorded, transcribed verbatim, and analyzed via dual-coder thematic analysis in accordance with COREQ standards. Saturation was reached when no new themes or codes emerged during review, and at least 2 anonymized supporting quotations per theme are reported for confirmability. Intercoder reliability was assessed, and transcript review was supplemented by member-checking for credibility. Reflexivity was maintained by both coders using analytic memos to document positionality and potential biases.

#### Data Analysis

##### Quantitative Analysis

Analyses were conducted in SPSS 26.0 (IBM Corp). Normality was assessed with the Shapiro-Wilk normality test. Between-group comparisons used 2-tailed independent-samples *t* tests for normally distributed variables and Mann-Whitney *U* tests otherwise. Two-tailed *α* was set at .05. We report test statistics, degrees of freedom, 95% CIs, and effect sizes (Cohen *d*) where applicable. Analyses followed a complete-case approach; the trial statistician was blinded to group assignment and had no role in data collection or intervention delivery.

##### Qualitative Analysis

Transcripts were analyzed using inductive thematic analysis in NVivo [55]. Two coders independently coded all transcripts; discrepancies were resolved via discussion with a third reviewer. Data saturation was deemed reached when no new codes/themes emerged. Trustworthiness was addressed through reflexive memos (researcher positionality); an audit trail; member-checking of summaries; and triangulation across parents, children, and clinicians. At least 2 anonymized quotations per theme are reported; intercoder reliability is provided.

## Results

### Result 1—Qualitative Needs Assessment: Barriers to Adherence and Design Motivators

#### Overview of Stakeholder Interviews

Table 2 shows a quantitative summary of stakeholder interview themes and subthemes from Stage 1 qualitative needs assessment in a 3-stage mixed methods study developing a gamified mobile intervention for pediatric amblyopia rehabilitation. Two overarching themes—Desires and Preferences—were derived through inductive thematic analysis with dual coding in NVivo 14 under COREQ-consistent procedures, and each subtheme is reported with participant proportion and an example code. Within Desires, adherence-relevant subthemes included sustained engagement (75%), enhancing the appeal of the eye patch (50%), rewards and immediate feedback (65%), and emotion regulation concerning feelings about being “in treatment,” “being ill,” and “wearing an eye patch” (95%). Within Preferences, children favored colorful collectibles (60%) and hands-on activities such as puzzles and drawing (45%). Interviews used purposive, maximum-variation sampling and included 8 pediatric ophthalmologists, 22 parents (8 families), and 6 children, conducted in clinic and child-friendly settings at the Eye & ENT Hospital of Fudan University.

The stakeholder interviews revealed a strong preference among parents and children for games that sustain attention over time. Key design motivators included continuous storylines; meaningful role-play; and clear, simple instructions—preferred over repetitive, single-player mini-games. These findings align with the high endorsement of narrative elements (75%) as reported in Table 2. Quantitative summary of theme frequencies, coded proportions, and representative quotations are presented to illustrate key patterns and their implications for therapy design.

**Table 2.** Quantitative results of stakeholder interviews.

Theme and subtheme	Participant proportion (%)	Code example
Desires		
The game needs to sustain attention	75	“Children need a continuous storyline”
Enhance appeal of the eye patch	50	“The eye patch causes discomfort”
Rewards mechanism	65	“Positive feedback motivates children”
Emotion regulation	95	“Reducing stigma, fear, and frustration” around “treatment/illness/eye-patch”
Preferences		
Preference for colorful items	60	“Children enjoy collecting colorful items”
Enjoyment of handicrafts	45	“Children like puzzles, drawing, etc”

#### Enhancing the Appeal of the Eye Patch

Families described both physical discomfort and peer-related embarrassment as major barriers to consistent patching (50%). Suggestions included attractive, customizable patterns and integrating patch-related actions with meaningful in-game events so that wearing the patch feels purposeful rather than punitive.

#### Rewards and Immediate Feedback

Parents reported that immediate, varied rewards are more effective than vague, delayed promises in sustaining daily participation (65%). Several children also mentioned striving for a high score as intrinsically motivating, while parents cautioned that repeating the same reward can lead to boredom.

#### Emotion Regulation

Interviews recurrently pointed to reducing stigma, fear, and frustration associated with treatment routines and visible patching (95% of parents). Typical comments included “If the patch could be part of the story—like a special pass to enter the game world—my child wouldn’t feel ‘sick’; she’d feel proud to wear it.” Parents also highlighted the need for gentle hints and affirming messages when children get stuck, and for empathetic narratives that normalize help-seeking and provide language to address teasing at school.

Mapped to cognitive appraisal theory, these requests correspond to valence (positive affect and feedback), agency (controllability through role-play), coping potential (scaffolding and hints), relevance (linking patch use to core game goals), and likelihood (clear goals and immediate feedback that reduce uncertainty).

#### Preferences

Under preferences, children spontaneously discussed liking bright, saturated colors (eg, pink, orange, blue), collectible items (stickers, stationery), and hands-on activities (puzzles, building, sketching). Many favored comic-style narratives and “becoming a character,” further supporting story-driven, role-based mechanics.

#### Design Recommendations

Synthesizing these findings, we propose 4 actionable recommendations for pediatric amblyopia training: (1) deliver quick, varied positive feedback to sustain engagement; (2) personalize content to children’s preferences and abilities; (3) embed rich storyworlds (animal companions, collectible artifacts) to deepen immersion; and (4) incorporate emotion-regulation affordances—empathetic cues, reframing of patching, and progressive challenges—to mitigate negative feelings surrounding treatment and illness and thereby strengthen adherence.

### Result 2—Qualitative Findings: Design Mapping and Prototype

#### Overview of Expert Interviews

Table 3 shows quantitative support from expert interviews in Stage 2, mapping the 5 cognitive appraisal dimensions to occlusion-therapy optimization and rehabilitation game-design principles for a gamified pediatric amblyopia intervention. This table summarizes expert-endorsed design mappings across Relevance, Valence, Likelihood, Agency, and Coping potential, reporting for each dimension the corresponding occlusion-therapy focus, game-design guideline, proportion of expert agreement, and an illustrative solution to support adherence. The expert process comprised 2 interview rounds with 6 experts (3 pediatric ophthalmologists and 3 game designers; ≥5 years’ experience), Zoom-based sessions (45-60 min), thematic coding with intercoder agreement κ=0.79, and an a priori consensus threshold of ≥80% on relevance and feasibility, informing Prototype 2 used in the RCT. Collectively, these principles specify how occlusion-therapy behaviors and game mechanics should be integrated to enhance adherence in pediatric amblyopia care.

**Table 3.** Quantitative support from expert interviews.

Appraisal dimension	Occlusion-therapy optimization	Rehabilitation training game design	Proportion (%)	Example of design solution
Relevance	Assign special meaning to the eye patch	Incorporate educational elements and understandable therapeutic goals	85	“Make friends with critters through treatment”
Valence	Link game progress with eye patch usage	Provide positive feedback and guidance	70	“Combines forms of visual training with therapeutic behaviors in play”
Likelihood	Showing the direct game effects of eye patch wear	Clear goal setting and feedback	65	“Unlock new maps after each level, add logos (corresponding eye sticker patterns) to the maps”
Agency	Emphasize the child’s role in the game through eye patches	Emphasize the leading role of children and role-playing	55	“Add interaction with animal companions”
Coping potential	Teach the correct use of eye patches	Emphasize progress rather than success	60	“Three achievement levels for each map”

#### Occlusion-Therapy Optimization

Experts recommended treating the eye patch as a meaningful artifact in the game world (eg, a narrative “pass” or “gift” from animal companions) to reframe patching as purposeful rather than punitive (Relevance). Progress in the game should depend visibly on correct patch use (Valence/Likelihood), and a short tutorial animation should demonstrate proper wear to reduce misuse and frustration (Coping potential).

#### Rehabilitation Game Mechanics (by Appraisal Dimension)

Rehabilitation game mechanics were mapped to the following appraisal dimensions:

- Relevance (85%): assign special meaning to the patch and incorporate clear therapeutic goals within the storyline.
- Valence (70%): deliver positive, specific feedback and guidance tied to therapeutic behaviors.
- Likelihood (65%): use explicit level goals and immediate feedback; successful sessions can unlock new maps and display patch-pattern “logos” on the map.
- Agency (55%): make the child the protagonist (eg, intern doctor), enable role-play, and add interaction with animal companions.
- Coping potential (60%): emphasize progress over perfection with 3 achievement tiers per map and adjustable difficulty to prevent overload.

#### Resulting Game Blueprint

**Figure 1.**
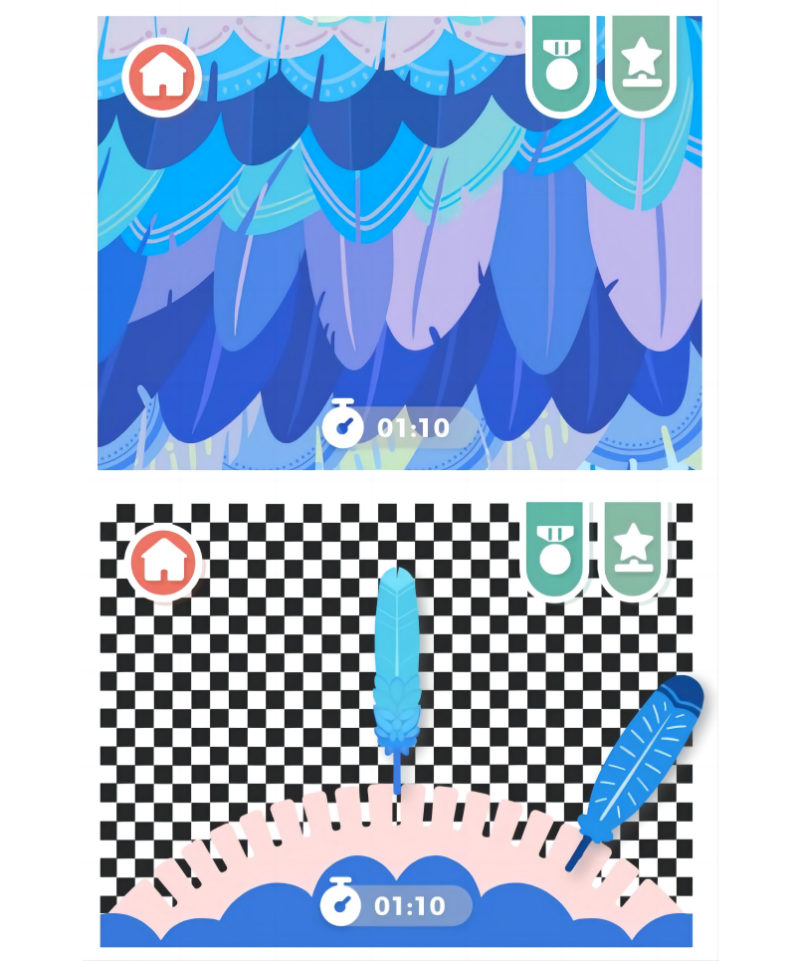
Visual stimulation training and fine eye training.

**Figure 2.**
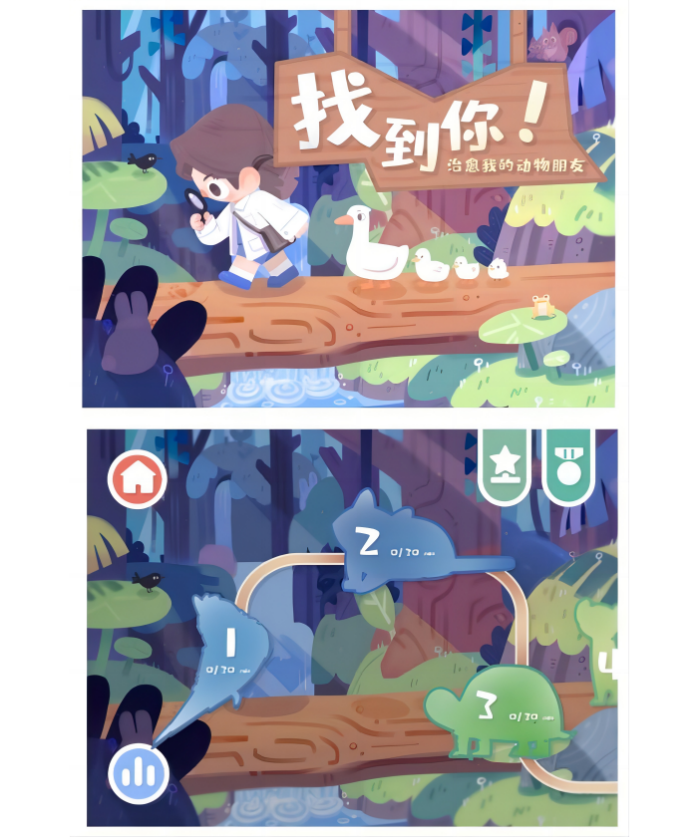
Main game interface and level interface.

Synthesizing these inputs, we adopted interactive storytelling as the primary format. Players act as an intern doctor who completes daily treatment tasks for animal patients (visual-training tasks embedded). The game includes 3 difficulty levels selectable by the child, collectible illustrations and achievements as incentives, and a child-friendly cartoon style. These principles were consolidated into a design-decision matrix and implemented in the intervention app used in Stage 3 (see Figures 1-2; Table 4).

**Table 4.** Participant characteristics.

	Experimental group, n (%)		Control group, n (%)	
Age (years)				
10	6 (33.33)		6 (37.5)	
9	7 (38.88)		6 (37.5)	
8	4 (22.22)		3 (18.75)	
7	1 (5.55)		1 (6.25)	
Sex				
Male	13 (72.22)		11 (68.75)	
Female	5 (27.77)		5 (31.25)	
Total	18 (100)		16 (100)	

#### Gamified Treatment App

The gamified mobile app “Find You! Cure My Animal Friends” developed for this study was designed to enhance treatment adherence in children with amblyopia by integrating cognitive appraisal theory. It provides a series of visually stimulating activities that are specifically crafted to improve visual acuity and encourage regular use, as shown in Figure 1. The main game interface and level interface are shown in Figure 2.

### Result 3—RCT Outcomes

#### User Statistics

Table 4 shows baseline characteristics of participants by randomized arm in the Stage 3 parallel-group RCT evaluating a gamified mobile app versus a commercial visual-training app for treatment adherence in pediatric amblyopia. Children aged 7-10 years were recruited via school outreach and clinic referrals in Shanghai, China, yielding an analytic sample of N=34 (intervention n=18; control n=16) after attrition. Variables include age distribution (7-10 y) and sex, with no statistically significant between-group differences at baseline.

The intervention and control groups yielded *W*=0.946 (*P*=.37) and *W*=0.963 (*P*=.71), respectively, indicating no evidence against normality. Normality assessment informed the use of independent-samples *t* tests alongside nonparametric tests in SPSS 26.0 with 2-tailed *α*=.05, as prespecified in the statistical analysis plan.

#### Adherence

After conducting an independent samples *t* test, a significant difference was found in the adherence scores on the MMAS-8 scale between the experimental group that used the amblyopia training game (*t*_32_=3.896; *P*<.001). The experimental group (N=18) had a mean adherence score of 6.56 (SD 1.06, SE 0.25, 95% CI 6.03-7.08), while the control group (N=16) had a mean score of 5.01 (SD 1.22, SE 0.31, 95% CI 4.36-5.66). The results indicated that the experimental group had significantly higher adherence scores than the control group.

Significant behavioral differences were found when comparing patient adherence to an amblyopia training game (experimental group) with other games on the market (control group). It was revealed that 61.11% (11/18) of patients in the experimental group showed high adherence, whereas only 18.75% (3/16) in the control group achieved the same level of adherence(*P*=.012). Additionally, the experimental group demonstrated a significantly lower percentage of patients with low adherence: 38.89% (7/18) in the experimental group versus 81.25% (13/16) in the control group(*P*=.012).

Table 5 shows the comparison of adherence issues between the intervention group and the control group. We provided the percentage of subjects who scored 0 for Q1-Q7, along with the mean score for Q8. The results revealed higher percentages in the control group for Q2, Q5, and Q7, which align with the significant differences observed in our statistical analyses. Significant differences emerged in some questions, notably “Have you missed any doses in the past two weeks?” (Q2: 11.11% vs 56.25%, *t_32_*=3.03; *P*<.001), indicating a higher compliance in the experimental group. Similarly, for “Did you find it challenging to adhere to the treatment program?” (Q7: 11.11% vs 50.00%, *t_32_*=2.59; *P*=.02), the experimental group showed significantly better adherence. No significant differences were observed between the experimental and control groups for questions about forgetting to take medication (Q1: 22.22% vs 31.25%, *t_32_*=0.58; *P*=.57), adjusting medication without informing the doctor (Q3: 11.11% vs 12.50%, *t_32_*=0.12; *P*=.90), and whether they took their medication the previous day (Q5: 11.11% vs 50.00%, *t_32_*=2.59; *P*=.02).

**Table 5.** Comparison of adherence issues between intervention and control groups.^a^

Questions	Experimental group (% or mean [SD])	Control group (% or mean [SD])	*t* test (*df*) or *z* statistic	*P* value
1. Do you ever forget to take your medication?	22.22%	31.25%	0.58(32)	.57
2. Have you missed any doses in the past two weeks?	11.11%	56.25%	3.03(32)	<*.001*
3. Do you adjust your medication without consulting your doctor when your symptoms worsen or new symptoms arise during treatment?	11.11%	12.50%	0.12(32)	.90
4. Do you sometimes forget to bring your medication with you when traveling or away from home for extended periods?	22.22%	25.00%	0.18(32)	.85
5. Did you take your medication yesterday?	11.11%	50.00%	2.59(32)	*.02*
6. Have you ever stopped taking your medication before completing the prescribed course, even if your symptoms improved or disappeared?	22.22%	25.00%	0.18(32)	.85
7. Did you find it challenging to adhere to the treatment program?	11.11%	50.00%	2.59(32)	*.02*
8. How frequently do you forget to take your medication?	0.67^b^ (0.27)	0.51^b^ (0.34)	−1.28^c^	.20

a Italic values indicate *P*<.05, showing statistical significance.

b Mean values.

c z-statistics.

#### User Experience Questionnaire

Table 6 shows the UEQ scores by randomized group at the 4-week assessment in the Stage 3 parallel-group RCT evaluating a gamified mobile app versus a commercial visual-training app for pediatric amblyopia rehabilitation (children aged 7-10 y, Shanghai, China, March-December 2024). The table reports means, standard deviations, sample sizes, and 95% CI for 6 UEQ dimensions (Attractiveness, Understandability, Efficiency, Reliability, Facilitation, and Novelty) in the intervention (n=18) and control (n=16) arms as a prespecified secondary outcome. The intervention outperformed the control on Attractiveness (2.14 vs 0.39), Understandability (1.88 vs 0.19), Facilitation (1.92 vs 1.14), and Novelty (2.13 vs 1.09), while the control showed a slight advantage on Efficiency (1.20 vs 1.08); Reliability was comparable (1.35 vs 1.30)

**Table 6.** User Experience Questionnaire scores.

Dimensions	Experimental group					Control group				
	Mean	SD	N	Confidence	95% CI	Mean	SD	N	Confidence	95% CI
Attractiveness	2.14	0.59	18	0.27	1.87 to 2.41	0.39	0.41	16	0.20	0.18 to 0.59
Understandability	1.88	0.78	18	0.36	1.51 to 2.24	0.19	0.57	16	0.28	–0.09 to 0.47
Efficiency	1.08	0.49	18	0.22	0.86 to 1.31	1.20	0.26	16	0.13	1.07 to 1.33
Reliability	1.35	0.62	18	0.29	1.06 to 1.63	1.30	0.23	16	0.11	1.19 to 1.41
Facilitation	1.92	0.45	18	0.21	1.71 to 2.13	1.14	0.26	16	0.13	1.01 to 1.27
Novelty	2.13	0.38	18	0.17	1.95 to 2.30	1.09	0.32	16	0.16	1.03 to 1.35

#### Personal Interviews

Table 7 shows postintervention individual interview results by randomized group mapping children’s experiences to cognitive appraisal dimensions in a 3-stage mixed methods study of a gamified mobile intervention for pediatric amblyopia rehabilitation (Stage 3 RCT, Shanghai, China, children aged 7-10 y). Interviews with children and parents were conducted via Zoom (20-40 min) after the 4-week intervention and thematically analyzed with dual-coder procedures consistent with COREQ, with codes organized under 3 themes: rehabilitation training experience, attitudes toward rehabilitation, and attitudes toward wearing eye patches. Codes are presented for the intervention and control groups and annotated with the corresponding appraisal dimension (relevance, valence, likelihood, agency, and coping potential) to show directional changes in perceived engagement and coping during treatment.

**Table 7.** Results of individual interviews.^a^

Theme	Experimental group (codes and dimensions)	Control group (codes and dimensions)
Rehabilitation training treatment experience	Children like the background of the story in the game (relevance↑) Children enjoy playing the role of a doctor (relevance↑, agency↑) Children enjoy interacting with animal friends in the game (relevance↑, agency↑) Children like the illustrations in the game to collect/unlock the list of friends (relevance↑, valence, likelihood↑)	Children find the mini-games a little boring (relevance↓) Children find the game graphics unattractive (valence↓) Children like getting high scores in games (relevance↑, likelihood↑)
Attitude toward rehabilitation training	Children like to play the game repeatedly to achieve high levels of achievement (relevance↑, coping potential↑, likelihood↑) Children perceive the game as difficult, but are more likely to want to complete the challenge (coping potential↑, likelihood↑) Children were still motivated to play the game at the end of the 30-day experiment (relevance↑, agency↑)	Children report resistance the first time they use the game (relevance↓, valence↓) Children say they are reluctant to repeat the training after experiencing all the mini-games (relevance↓) Children play the same game 2-‐3 times to get a high score (relevance↑, likelihood↑, coping potential↑)
Attitude toward wearing eye patches	Children are generally motivated to wear eye patches (relevance↑, agency↑) Children like eye patches with special patterns and keep them carefully (relevance↑, valence↑)	Children are often resistant to the eye patch and require parental intervention (valence↓, coping potential↓)

a “↑” indicates an increase or improvement, while “↓” signifies a decrease or reduction.

The study found that children in the experimental group had a positive reaction to the gamified rehabilitation training. This positive response may be attributed to the game design being consistent with cognitive appraisal theory. Children particularly enjoyed storytelling, role-playing, interacting with in-game animal characters, and collecting icons. These elements increased their perceptions of goal relevance and congruence, as well as their assessments of the likelihood of successfully completing the game. Furthermore, enabling the children to actively engage in the game and interact with the surroundings enhanced their sense of control and ability to cope, which could account for the experimental group’s greater participation in the game and adherence to rehabilitation training. The experiences and attitudes of the children in the control group differed significantly, as they mostly expressed disinterest in the game content or found the images unattractive. However, even in the control group, children were willing to play the game repeatedly to achieve a high score, indicating that perceptions of likelihood and coping potential can still be improved with appropriate incentives. Furthermore, the experimental group’s children exhibited a higher level of positive acceptance toward the specially patterned eye patches, which provides additional evidence that increasing goal relevance and consistency can enhance treatment adherence. Conversely, the control group’s children were less receptive to eye patches and needed parental intervention.

## Discussion

### Principal Results and Contextualization

This study explored a gamified mobile app developed with cognitive appraisal theory to improve treatment adherence among children with amblyopia. Compared with a commercially available alternative, our app significantly improved self-reported adherence and user engagement. These findings resonate with prior research showing that gamified interventions tailored to pediatric needs can increase adherence, particularly when emotional engagement and role-play are incorporated into therapeutic design [58]. Our findings reinforce this, demonstrating that when visual therapy is embedded within a child-centric narrative, such as acting as a doctor for animal patients, treatment becomes more intrinsically motivating and socially meaningful.

Prior digital therapies for amblyopia often emphasized perceptual gains while overlooking emotional dimensions, limiting their long-term efficacy [32,44]. In contrast, our results suggest that integrating emotional resonance through personalized narratives enhances not only short-term adherence but also sustained behavioral engagement. Thematic interview results indicate children developed emotional attachment to in-game characters, aligning with self-determination theory, which emphasizes autonomy, competence, and relatedness as key drivers of motivation [59]. These findings bridge cognitive appraisal theory with game-based learning literature, supporting an integrative framework for digital therapeutic design.

The app’s ability to improve perceived coping potential—a critical appraisal dimension—suggests that such designs may empower children by increasing their perceived control and self-efficacy. This finding aligns with pediatric psychology literature, where increased agency and coping predict better adherence in chronic treatment contexts [60]. Similar conclusions have been drawn in pediatric diabetes and asthma care, where gamified self-management tools improved perceived autonomy and long-term outcomes [61,62]. Our results extend these insights to amblyopia care, suggesting cross-condition applicability of emotionally intelligent gamification.

In addition to cognitive appraisal theory, the positive reception of the product aligns with established game engagement models, such as the Player Experience of Need Satisfaction model, which has been shown to predict retention and satisfaction in health games [63]. The high UEQ scores in attractiveness and novelty observed in our trial mirror predictors of engagement identified in broader mobile health research [64]. Together, these results underscore the value of designing from a multidimensional framework that spans affective science, pediatric behavioral health, and human-computer interaction.

### Implications for Practice and Design

These findings have several implications for both clinical practice and digital health design. First, clinicians should consider recommending therapeutic applications that support emotional regulation and engagement, not just visual training efficacy. Second, designers should consider embedding rehabilitation goals within game narratives that emphasize positive emotions, role-play, and intrinsic reward mechanisms. Our approach could be adapted to other chronic pediatric conditions requiring repetitive behavior change, such as speech therapy or motor training.

Additionally, co-design with children and caregivers is essential to ensure developmental appropriateness and motivational alignment. Our use of animal avatars, customizable achievements, and therapeutic storytelling was informed by early-stage qualitative interviews, reinforcing the value of participatory design in digital health tool development [65].

### Future Research Directions

Longer follow-up trials are needed to determine whether appraisal-informed gamified therapy produces sustained improvements in visual function (eg, visual acuity and binocular outcomes) beyond short-term adherence gains, given the current paucity of long-term evidence for digital interventions in amblyopia. Future studies should incorporate objective adherence metrics (eg, standardized usage logs in both arms) and conduct deeper psychometric evaluation of the adapted MMAS-8, including test-retest reliability, Rasch calibration, and convergent validity against behavioral data, to address limitations of self-reports and strengthen measurement robustness. Multisite and larger-sample RCTs with cross-cultural recruitment are warranted to assess generalizability of emotionally adaptive gamification across diverse pediatric populations and health care settings. Designing and testing explicit parent- or family-centered coengagement components may further improve adherence, building on evidence that family involvement and supportive feedback can enhance therapeutic participation in pediatric amblyopia. Mechanistic studies should isolate how specific appraisal dimensions (relevance, valence, likelihood, agency, coping potential) drive adherence and engagement to optimize narrative, feedback, and role-play elements in serious games for vision rehabilitation. Safety evaluations should also monitor potential adverse effects related to screen exposure (eg, visual fatigue or myopia risk) to inform risk-benefit profiles for home use of small-screen devices in pediatric care. Finally, integration of occlusion-therapy behaviors with in-game mechanics should be quantitatively linked to clinical endpoints (eg, patch-wear patterns and acuity change) to clarify therapeutic synergy in real-world home settings.

### Limitations

This study has several limitations. First, the sample size was modest and drawn from a limited setting, which may affect generalizability. Second, the evaluation focused on short-term adherence (4 weeks); the sustainability of effects and links to long-term visual outcomes require follow-ups. Third, while the adapted MMAS-8 offered a brief and accepted measure for pediatric behavioral adherence, its origin in medication contexts introduces potential semantic transfer; although cognitive interviewing and a pilot supported face validity and internal consistency (*α*=.83), further psychometric work (eg, Rasch calibration, test-retest, and convergent validity against usage logs and visual-acuity change) is warranted. Fourth, adherence included self-reports, which are susceptible to recall and social-desirability bias. The comparable logs were unavailable for the commercial control app, limiting between-arm objective comparisons. Finally, the game primarily targeted child users; although parents supported use, the intervention did not explicitly structure parental coengagement, which is known to influence adherence and could be incorporated in future iterations.

### Conclusion

This 3-stage mixed methods study developed and evaluated a cognitive appraisal–informed mobile serious game to support home-based rehabilitation for pediatric amblyopia, with a prespecified primary focus on treatment adherence and secondary assessments of user experience and qualitative engagement. In a 4-week parallel-group RCT, the intervention significantly improved adherence on the adapted MMAS-8 when compared with a commercial control app (mean 6.56 vs 5.01; *t*_35_=3.896; *P*<.001), with a higher proportion of high-adherence participants and fewer participants with low adherence in the intervention arm. Secondary outcomes showed superior user experience for the intervention in terms of attractiveness, understandability, facilitation, and novelty, which is consistent with stronger engagement profiles for pediatric digital health tools. Posttrial interviews mapped children’s experiences to appraisal dimensions, indicating higher relevance, valence, likelihood, agency, and coping potential in the intervention group alongside more positive attitudes toward patch use, aligning with the study’s theory-driven design. Collectively, these findings demonstrate that an appraisal-guided, child-centric gamified intervention can enhance short-term adherence and user experience in home amblyopia care, directly meeting the stated study aim of supporting sustained home-based rehabilitation through emotionally intelligent digital therapy.

## Supplementary material

10.2196/60309 Multimedia Appendix 1 Interview outline for parents (preintervention).

10.2196/60309 Multimedia Appendix 2 Children’s interest and preference questionnaire.

10.2196/60309 Multimedia Appendix 3 Interview outline for medical experts.

10.2196/60309 Multimedia Appendix 4 Adapted 8-item Morisky Medication Adherence Scale (MMAS-8).

10.2196/60309 Multimedia Appendix 5 User Experience Questionnaire (UEQ).

10.2196/60309 Multimedia Appendix 6 Postintervention interview outline for parents.

10.2196/60309 Checklist 1 COREQ 32-item checklist.

10.2196/60309 Checklist 2 CONSORT 2010 checklist of information to include when reporting a randomized trial.

## Abbreviations

CONSORT-EHEALTH: Consolidated Standards of Reporting Trials of Electronic and Mobile Health Applications and Online Telehealth
COREQ: Consolidated Criteria for Reporting Qualitative Studies
MMAS-8: 8-item Morisky Medication Adherence Scale
RCT: randomized controlled trial
UEQ: User Experience Questionnaire
