# Supplementary material for: Effectiveness of a Gamified Mobile App in Enhancing Treatment Adherence for Children With Amblyopia: Explorative Study
Source: JMIR Serious Games. 2025 Oct 28;13:e60309. doi: 10.2196/60309 (PMC12569704; doi:10.2196/60309)
Supplement: Multimedia Appendix 1 [file games-v13-e60309-s001.docx]

**Multimedia Appendix 1: Interview Outline for Parents (Pre-Intervention)**

1. Purpose of the Interview:

To understand parents' perspectives on adherence to amblyopia treatment for children and the potential impact of a gamified training program in the home environment.

2. Duration of the Interview:

30–45 minutes

3. Format of the Interview:

Semi-structured interview

4. Preparation Tools:

Audio recorder, printed outline, informed consent form, basic information sheet

5. Location of the Interview:

Shenzhen Children's Hospital

6. Selection of Interview Subjects:

Parents of children aged 6–10 years diagnosed with anisometropic amblyopia

7. Interview Content:

Basic Information

How old is your child?

When and how did you discover that your child had amblyopia?

How long has your child been undergoing amblyopia rehabilitation training? What is the training frequency?

Understanding of Amblyopia

How familiar are you with amblyopia? Do you know what kind of eye condition it is?

How long do you think rehabilitation training needs to continue for ideal vision improvement?

Challenges and Support in Amblyopia Training

Before starting app-based training, how did you assist your child with amblyopia treatment?

Were there any memorable events or challenges during treatment?

What challenges did you encounter in supporting your child’s treatment? What motivated you to keep going?

Behavioral Characteristics of Children with Amblyopia

Do you think your child faces any inconveniences in daily life due to amblyopia?

Are you aware of your child’s performance at school? Is it different from their behavior at home?

How do you usually help your child manage amblyopia symptoms?

Attitudes and Views on the Gamified Training Program

What aspects do you hope to improve in your child through rehabilitation training?

How interested is your child in app-based training? Has their willingness to train changed?

In your opinion, what are the key elements that should be included in a gamified amblyopia rehabilitation program to help children adhere to the training?

What is your opinion on using an iPad for amblyopia rehabilitation training? Do you think this method could increase your child's enthusiasm?
