# Supplementary material for: Effectiveness of a Gamified Mobile App in Enhancing Treatment Adherence for Children With Amblyopia: Explorative Study
Source: JMIR Serious Games. 2025 Oct 28;13:e60309. doi: 10.2196/60309 (PMC12569704; doi:10.2196/60309)
Supplement: Multimedia Appendix 2 [file games-v13-e60309-s002.docx]

**Multimedia Appendix 2: Children’s Interest and Preference Questionnaire**

1. Purpose of the Questionnaire:

To understand children's interests and preferences, especially regarding game-related activities.

2. Expected Number of Responses:

50

3. Questionnaire Location:

Shenzhen Children's Hospital

4. Target Respondents:

Children aged 6–10 years accompanied by parents

5. Questionnaire Content:

Personal Information

How old are you?

What grade are you currently in?

Color Preference

What is your favorite color?

Interest in Activities and Game Preferences

Do you attend any extracurricular classes? If so, what type of class?

What is your favorite activity?

What game do you like to play the most?

Have you played any video games? If so, which ones?

Preferences for Cartoons and Characters

What is your favorite toy?

What is your favorite cartoon show?

Who is your favorite cartoon character?

Usage of Electronic Devices

What electronic devices do you often use at home?

How long do you usually use electronic devices each time?
