# Supplementary material for: Effectiveness of a Gamified Mobile App in Enhancing Treatment Adherence for Children With Amblyopia: Explorative Study
Source: JMIR Serious Games. 2025 Oct 28;13:e60309. doi: 10.2196/60309 (PMC12569704; doi:10.2196/60309)
Supplement: Multimedia Appendix 3 [file games-v13-e60309-s003.docx]

**Multimedia Appendix 3: Interview Outline for Medical Experts**

1. Purpose of the Interview:

To gather doctors' perspectives on interventions for pediatric amblyopia, understand the current standard treatment methods, identify challenges in adherence, and collect suggestions for gamified therapeutic approaches.

2. Duration of the Interview:

15–60 minutes

3. Format of the Interview:

Combination of in-person and online semi-structured interviews

4. Preparation Tools:

Audio recorder, printed outline

5. Location of the Interview:

Fudan University Affiliated Hospital, Shanghai

6. Interview Content:

What methods do you typically recommend for treating amblyopia in children?

What medical guidelines need to be followed when treating pediatric amblyopia?

What adherence challenges are encountered with current treatment methods?

What are your views on the practical application of gamified training in amblyopia treatment for children?

What factors do you think could improve treatment adherence in children?

Which elements in gamified design (e.g., reward mechanisms) do you believe could better engage children in their treatment?
