# Supplementary material for: Effectiveness of a Gamified Mobile App in Enhancing Treatment Adherence for Children With Amblyopia: Explorative Study
Source: JMIR Serious Games. 2025 Oct 28;13:e60309. doi: 10.2196/60309 (PMC12569704; doi:10.2196/60309)
Supplement: Multimedia Appendix 4 [file games-v13-e60309-s004.docx]

**Multimedia Appendix 4: Adapted MMAS-8**

| Adherence to Amblyopia Training (Adapted MMAS-8;Caregiver-Assisted) | | |
| --- | --- | --- |
| Instructions:The questions below ask about your child’s home vision training using the study game and wearing the eye patch as instructed by your clinician. There are no right or wrong answers. Please think about your child’s usual routine (and the last two weeks where specified) and answer each item.Please tick one option for Items 1–7 (Yes/No). For Item 8, circle one choice (a-e). | | |
| 1.Do you sometimes forget for your child to **do the vision training** as scheduled? | Yes | No |
| 2.Thinking over the **past two weeks**, were there any days when your child **did not do the vision training**, for reasons other than forgetting? |  |  |
| 3.Have you ever **cut back or stopped** your child’s training **without telling the doctor/therapist**, because your child felt worse or uncomfortable (e.g., eye strain, irritation) when doing it? |  |  |
| 4.When you **travel or are away from home**, do you sometimes forget to bring what is needed for training (e.g., **eye patch/device**) so the session is missed? |  |  |
| 5.Did your child **do the vision training yesterday** (or the **last time** it was scheduled)? |  |  |
| 6.When you feel your child’s **vision seems under control or improving**, do you sometimes **stop or reduce** the training? |  |  |
| Doing training every day can be inconvenient for some families. Do you ever feel **hassled** about sticking to your child’s **training plan**? |  |  |
| ****How often**** do you/your child have difficulty ****remembering to complete all parts**** of the training (e.g., ****wear the eye patch as instructed and finish the full session time****)? | | |
| a) **Never/Rarely** b) **Once in a while** c) **Sometimes** d) **Usually** e) **All the time** | | |
