# Supplementary material for: Effectiveness of a Gamified Mobile App in Enhancing Treatment Adherence for Children With Amblyopia: Explorative Study
Source: JMIR Serious Games. 2025 Oct 28;13:e60309. doi: 10.2196/60309 (PMC12569704; doi:10.2196/60309)
Supplement: Multimedia Appendix 5 [file games-v13-e60309-s005.docx]

**Multimedia Appendix 5: User Experience Questionnaire (UEQ)**

Instructions:

Hello, and thank you for participating in this experiment! We hope you can share your honest experience with this product.

Please fill out the following questionnaire to evaluate the user experience of this product. The questionnaire consists of 26 pairs of opposite adjectives, each describing an aspect of the product. Between each pair of adjectives is a 7-point rating scale, with each level represented by a circle. Please select the circle that best reflects the extent to which the product aligns with each adjective.

Example:

Enjoyable ⚪ ⚪ ⚪ ⚪ ⚪ ⚪ ⚪ Boring

Check the circle that best reflects your true impression. Please answer intuitively without overthinking, as this will best capture your first impressions. Some pairs may not completely match your feelings, but please choose an option that best describes your view. There are no right or wrong answers, as we value your personal opinion!

Questionnaire Content:

Please rate the following adjective pairs:

Enjoyable ⚪ ⚪ ⚪ ⚪ ⚪ ⚪ ⚪ Boring

Easy to understand ⚪ ⚪ ⚪ ⚪ ⚪ ⚪ ⚪ Difficult

Engaging ⚪ ⚪ ⚪ ⚪ ⚪ ⚪ ⚪ Uninteresting

Useful ⚪ ⚪ ⚪ ⚪ ⚪ ⚪ ⚪ Useless

Friendly ⚪ ⚪ ⚪ ⚪ ⚪ ⚪ ⚪ Cold

Efficient ⚪ ⚪ ⚪ ⚪ ⚪ ⚪ ⚪ Inefficient

Reliable ⚪ ⚪ ⚪ ⚪ ⚪ ⚪ ⚪ Unreliable

Innovative ⚪ ⚪ ⚪ ⚪ ⚪ ⚪ ⚪ Conventional

Simple ⚪ ⚪ ⚪ ⚪ ⚪ ⚪ ⚪ Complex

Pleasant ⚪ ⚪ ⚪ ⚪ ⚪ ⚪ ⚪ Frustrating

Intuitive ⚪ ⚪ ⚪ ⚪ ⚪ ⚪ ⚪ Confusing

Lively ⚪ ⚪ ⚪ ⚪ ⚪ ⚪ ⚪ Dull

Valuable ⚪ ⚪ ⚪ ⚪ ⚪ ⚪ ⚪ Worthless

Interesting ⚪ ⚪ ⚪ ⚪ ⚪ ⚪ ⚪ Dull

Trustworthy ⚪ ⚪ ⚪ ⚪ ⚪ ⚪ ⚪ Untrustworthy

Attractive ⚪ ⚪ ⚪ ⚪ ⚪ ⚪ ⚪ Ugly

Flexible ⚪ ⚪ ⚪ ⚪ ⚪ ⚪ ⚪ Rigid

Fast ⚪ ⚪ ⚪ ⚪ ⚪ ⚪ ⚪ Slow

Easy to learn ⚪ ⚪ ⚪ ⚪ ⚪ ⚪ ⚪ Hard to learn

Feels safe ⚪ ⚪ ⚪ ⚪ ⚪ ⚪ ⚪ Unsafe

Inspiring ⚪ ⚪ ⚪ ⚪ ⚪ ⚪ ⚪ Disappointing

Satisfying ⚪ ⚪ ⚪ ⚪ ⚪ ⚪ ⚪ Unsatisfying

Creative ⚪ ⚪ ⚪ ⚪ ⚪ ⚪ ⚪ Uncreative

Fun ⚪ ⚪ ⚪ ⚪ ⚪ ⚪ ⚪ Dull

Secure ⚪ ⚪ ⚪ ⚪ ⚪ ⚪ ⚪ Insecure

Easy to use ⚪ ⚪ ⚪ ⚪ ⚪ ⚪ ⚪ Hard to use

Thank you for your participation and feedback!
