# Supplementary material for: Effectiveness of a Gamified Mobile App in Enhancing Treatment Adherence for Children With Amblyopia: Explorative Study
Source: JMIR Serious Games. 2025 Oct 28;13:e60309. doi: 10.2196/60309 (PMC12569704; doi:10.2196/60309)
Supplement: Multimedia Appendix 6 [file games-v13-e60309-s006.docx]

**Multimedia Appendix 6: Post-Intervention Interview Outline for Parents**

1. Purpose of the Interview:

To gather feedback based on the training experience, understand the psychological impact on participants post-intervention, and identify reasons for their likes or dislikes. Additionally, to obtain parents' opinions and suggestions regarding the design of the intervention product for anisometropic amblyopia training.

2. Interview Content:

How did your child feel about the product used in the experiment? Did they share any thoughts with you?

Did your child experience any discomfort, or display any noticeable behaviors or reactions?

If we were to further improve this type of anisometropic amblyopia training product, would you consider having your child use it again?

What are your thoughts and suggestions on the intervention product used in this experiment? Are there any areas that could be optimized or improved?
